# A CT-based radiomics predictive nomogram to identify pulmonary tuberculosis from community-acquired pneumonia: a multicenter cohort study

**DOI:** 10.3389/fcimb.2024.1388991

**Published:** 2024-09-19

**Authors:** Pulin Li, Jiling Wang, Min Tang, Min Li, Rui Han, Sijing Zhou, Xingwang Wu, Ran Wang

**Affiliations:** ^1^ Department of Respiratory and Critical Care Medicine, The First Affiliated Hospital of Anhui Medical University, Hefei, China; ^2^ Department of Infectious Disease, Hefei Second People’s Hospital, Hefei, China; ^3^ Department of Oncology, The First Affiliated Hospital of Anhui Medical University, Hefei, China; ^4^ Department of Occupational Disease, Hefei Third Clinical College of Anhui Medical University, Hefei, China; ^5^ Department of Radiology, The First Affiliated Hospital of Anhui Medical University, Hefei, China

**Keywords:** pulmonary tuberculosis, community-acquired pneumonia, computed tomography, radiomics, nomogram

## Abstract

**Purpose:**

To develop a predictive nomogram based on computed tomography (CT) radiomics to distinguish pulmonary tuberculosis (PTB) from community-acquired pneumonia (CAP).

**Methods:**

A total of 195 PTB patients and 163 CAP patients were enrolled from three hospitals. It is divided into a training cohort, a testing cohort and validation cohort. Clinical models were established by using significantly correlated clinical features. Radiomics features were screened by the least absolute shrinkage and selection operator (LASSO) algorithm. Radiomics scores (Radscore) were calculated from the formula of radiomics features. Clinical radiomics conjoint nomogram was established according to Radscore and clinical features, and the diagnostic performance of the model was evaluated by receiver operating characteristic (ROC) curve analysis.

**Results:**

Two clinical features and 12 radiomic features were selected as optimal predictors for the establishment of clinical radiomics conjoint nomogram. The results showed that the predictive nomogram had an outstanding ability to discriminate between the two diseases, and the AUC of the training cohort was 0.947 (95% CI, 0.916-0.979), testing cohort was 0.888 (95% CI, 0.814-0.961) and that of the validation cohort was 0.850 (95% CI, 0.778-0.922). Decision curve analysis (DCA) indicated that the nomogram has outstanding clinical value.

**Conclusions:**

This study developed a clinical radiomics model that uses radiomics features to identify PTB from CAP. This model provides valuable guidance to clinicians in identifying PTB.

## Introduction

Tuberculosis is a chronic respiratory infection caused by Mycobacterium tuberculosis, among which pulmonary tuberculosis (PTB) is the most common, accounting for more than 80% of tuberculosis (Dheda et al., 2016; Drain et al., 2018). In general, the early symptoms of PTB are not obvious and often go unnoticed, thus accelerating the progress of the disease. Therefore, early diagnosis is crucial to the treatment of tuberculosis (Walzl et al., 2018). At present, the diagnosis of tuberculosis mainly depends on sputum smear and culture, and the gold standard for diagnosis is sputum bacteriological detection. However, bacteriological detection of sputum takes longer time, usually more than four weeks (Schito et al., 2015; Suárez et al., 2019). As a result, the source of infection cannot be controlled, leading to an increase in the incidence of tuberculosis and mortality. Secondary tuberculosis is the most common type of PTB in adults, and its imaging manifestations are varied and often confused with other pulmonary infections, especially community-acquired pneumonia (CAP) (Lyon and Rossman, 2017; Nachiappan et al., 2017).

CAP is pneumonia acquired outside a hospital. The most common pathogens are Streptococcus pneumoniae, Hemophilus Influenza, and atypical bacteria (e.g. Chlamydia pneumoniae, Mycoplasma pneumoniae, Legionella, and the virus). CAP often manifests as fever, cough, excessive phlegm, chest pain, dyspnea, tachypnea, and tachycardia. Secondary tuberculosis also has some of the same symptoms (Qi et al., 2021), but they are treated with different drugs (Modi and Kovacs, 2020; Peloquin and Davies, 2021). The incidences of PTB and CAP in the world is increasing year by year, and many patients are not treated in time, often resulting in serious complications. Therefore, a simple and noninvasive method to quickly distinguish PTB from CAP is necessary for later treatment of the disease.

Radiomics is a computational, noninvasive method for extracting quantitative features from images and transforming the information into a mineable database (Mayerhoefer et al., 2020). Radiomics can provide multitudes of information that cannot be found by the naked eye, and can sensitively judge the subtle changes of morphology and function in different parts of the lesion (Moon et al., 2019; Han et al., 2023). This approach has been widely used not only in lung cancer (He et al., 2020; Kirienko et al., 2021; Tunali et al., 2021), but now in COVID-19 as well (Bouchareb et al., 2021). but it is seldom studied in PTB and CAP.

Our study aims to develop a predictive nomogram based on computed tomography (CT) radiomics. It is used early to distinguish PTB from CAP so that these patients can receive timely treatment.

## Method

### Patients

We collected patients with pulmonary tuberculosis or community-acquired pneumonia from June 2018 to May 2023 from three medical centres. There were 195 PTB patients and 163 CAP patients who met the inclusion and exclusion criteria. Clinical characteristics collected included sex, age, white blood cell count (WBC), neutrophil ratio (NEUT%), neutrophil to lymphocyte ratio (LYMPH%), C-reactive protein (CRP), Hemoglobin (HGB), platelets (PLT), albumin (ALB), glucose (GLU), T-SPOT result, fever (body temperature ≥37.3°C), cough, expectoration, and hemoptysis. This retrospective study was approved by the Research Ethics Committee of the First Affiliated Hospital of Anhui Medical University (Hospital I), Hefei Second People’s Hospital (Hospital II) and the First Affiliated Hospital of Soochow University (Hospital III).

#### Inclusion criteria

1.Patients with PTB and CAP, and CT imaging data before anti-tuberculosis and anti-infective treatment; 2. The CT imaging data is clear and can be extracted for interpretation. 3. There was no complication of respiratory diseases with pulmonary imaging features (such as lung cancer, interstitial lung disease, and so on).

#### Exclusion criteria

1. Under 18 years of age; 2. Insufficient clinical information to provide; 3. CT showed no obvious lung lesions.

#### Diagnostic criteria for patients with PTB

Patients with PTB were diagnosed with having a positive smear or tuberculosis culture in at least one respiratory tract sample (sputum or alveolar lavage fluid or lung puncture fluid).

#### Diagnostic criteria for patients with CAP

Adults over 18 years of age with clinical signs suggestive of CAP, acquired outside the hospital or less than 48 hours after admission, meet CAP criteria (Cao et al., 2018).

### CT examinations

All enrolled patients underwent plain chest CT scans before antibiotic or anti tuberculous medication. The CT scanning equipment and parameters of the three hospitals are shown in supplementary document **Supplementary Table S1**. Scan range from the apex of both lungs to the bottom of the lungs. All chest CT parameters were fixed according to the guidelines of the CT scanning protocol.

### CT image segmentation and radiomic feature extraction

A radiologist with 10 years of experience in radiology used ITK-SNAP 3.8.0 (ITK-SNAP Home (itksnap.org)) manually segmented the lung window of all CT images. The same window parameters were selected for all CT images (window width 2000HU, window level-400HU). Because of the diffuse and multiple manifestations of inflammatory lesions, it is difficult to accurately delineate all lesions. Therefore, we selected the maximum level of ground-glass attenuation and outlined all the lesions in this level as a volume of interest (VOI), then sketch layer by layer to form the 3D VOI. Another radiologist with more than 10 years of experience reviewed the imaging features of the lesion as well as the VOI. The two radiologists worked independently. The consistency and repeatability of the results were evaluated by inter-class and intra-class correlation coefficients (ICCs). The higher the ICC value, the higher the reproducibility. Features with ICC below 0.75 are considered to have poor feature consistency, so they will be removed. Before extracting radiomic features, all images were resampled and image normalized to achieve zero mean and unit variance (Zhu et al., 2021; Yang et al., 2022). All radiomics features were extracted from VOIs using PyRadiomics (van Griethuysen et al., 2017).

### Radiomic signature construction

First, the dataset of hospital I and hospital II is randomly assigned to the training and testing cohort in a 8:2 ratio, and the dataset of hospital III is validation cohort. The training cohort is used to predict the model. The testing cohort and validation cohort are used to independently evaluate the performance of the model. Finally, Z-score is used to standardize the training cohort data, and use it to normalize the test and validation cohorts. Then, the features with ICCs >0.75 were retained, and the features with P <0.05 were screened by univariate logistic analysis. A least absolute shrinkage and selection operator (LASSO) and multivariate logistic analysis were used for further screening, and independent risk predictors were retained (P <0.05). Finally, Radscore was calculated by using the formula based on radiomics characteristics. We used the support vector machine (SVM) algorithm to construct the radiomics prediction model. SVM is a large-margin classification model. Its basic model is a linear classifier defined in the feature space with the maximum interval (Valkenborg et al., 2023).

### Construction of the clinical model

Univariate analysis was performed according to the included clinical characteristics with CAP and PTB in training cohort. Multivariate logistic regression analysis was performed for the clinical factors with P < 0.05. The clinical model was predicted according to the results of multivariate logistic regression analysis (P < 0.05) and variance inflation factor.

### Establishment of a clinical radiomics conjoint nomogram

The clinical radiomics conjoint nomogram was constructed by combining the clinical factors related to the clinical model with Radscore in the radiomics model. receiver operating characteristic (ROC) curve and Decision curve analysis (DCA) were used to compare the diagnostic and predictive performance of the clinical model, radiomics model, and clinical radiomics conjoint model in the training and test cohorts.

### Statistical analyses

All radiomics analyses were conducted using Python version 3.5.6. Statistical analysis was performed using R 4.1.2 (R: The R Project for Statistical Computing (r-project.org)). Continuous variables with normal distribution were expressed as mean ± standard deviation. Data that are not normally distributed were expressed as median (range interquartile) and analyzed using the rank-sum test. Independent samples t-test or Mann-Whitney U test were used to compare continuous variables. The Chi-square test or Fisher exact test was used for categorical variables. A two-sided p-value < 0.05 was used to indicate statistical significance.

## Results

### Basic clinical characteristics

After the screening, a total of 358 patients were included in this study, including 195 PTB patients and 163 CAP patients. Among them, 170 patients were trained, 73 patients were tested and 115 patients were verified. The study flow diagram is shown in **Supplementary Figure S1**. **Table 1** shows the clinical features of patients. In the all cohort, univariate and multivariate logistic regression showed that T-SPOT.TB result (P < 0.001) and fever (P < 0.001) were two independent predictors in the clinical model (**Supplementary Table S2**).

**Table 1:** Clinical features of Patients with PTB and CAP.

features	All Cohort		Training Cohort		Testing Cohort		Validation Cohort	
	CAP	PTB	CAP	PTB	CAP	PTB	CAP	PTB
n	163	195	74	96	32	41	58	57
Sex (%)								
Female	86 (24%)	79 (22.1%)	45 (26.5%)	40 (23.5%)	12 (16.4%)	11 (15.1%)	30 (26.1%)	28 (24.3%)
Male	77 (21.5%)	116 (32.4%)	29 (17.1%)	56 (32.9%)	20 (27.4%)	30 (41.1%)	28 (24.3%)	29 (25.2%)
Age	51 (31, 67)	61 (46, 71.5)	49 (30.25, 66)	62 (41, 73)	52 (35.75, 66.25)	64 (49, 70)	60 (42.25, 69.75)	52 (31, 67)
WBC	6.26 (4.93, 8.20)	7.19 (5.54, 9.36)	6.26 (4.51, 8.70)	7.20 (5.57, 9.35)	5.97 (5, 7.44)	6.92 (4.99, 8.97)	7.64 ± 2.92	7.12 ± 2.88
NEUT%	64.2 (55.8, 74.75)	72.64 (63.95, 81.01)	64.49 ± 14.63	71.38 ± 11.92	64.32 ± 12.17	72.81 ± 11.96	72.26 ± 10.70	66.53 ± 14.73
LYMPH%	25.8 (16.35, 32.15)	15.54 (11.4, 24.75)	24.9 (16.16, 35.06)	16.5 (11.65, 24.88)	27.3 (20.49, 31.83)	14.9 (8.8, 24.7)	15.52 (11.55, 24.48)	23.5 (14.84, 31)
HGB	122 (110.5, 134)	114 (100.5, 125)	120.5 (111, 131)	114.5 (101, 126.25)	125.47 ± 16.629	114.12 ± 20.44	113 (98, 122.5)	124 (110, 134)
PLT	217 (183, 290)	247 (204.5, 334)	215.5 (174.25, 290)	233.5 (195.5, 318.25)	218.56 ± 56.786	288.9 ± 126.4	254 (221, 337.75)	235 (196, 303)
ALB	37.93 ± 6.17	34.86 ± 5.63	38.51 ± 6.01	35.11 ± 5.18	38.4 (34.28, 41.53)	33.9 (30.4, 37.9)	35.10 ± 5.81	37.37 ± 6.48
GLU	5.33 (4.73, 6.04)	5.06 (4.56, 5.79)	5.33 (4.65, 6.14)	4.985 (4.54, 5.54)	5.21 (4.76, 5.77)	5.12 (4.56, 6.13)	5.09 (4.6, 5.76)	5.41 (4.97, 6.17)
CRP	22.1 (2.81, 76.89)	28.2 (7.45, 70.05)	18.19 (2.98, 66.04)	28.78 (6.30, 54.83)	16.4 (1.35, 61.36)	24.4 (7.4, 77.7)	28.64 (9.5, 71.95)	36.1 (3.44, 83.1)
T-SPOT, (%)								
negative	129 (36%)	42 (11.7%)	62 (36.5%)	19 (11.2%)	23 (31.5%)	7 (9.6%)	16 (13.9%)	44 (38.3%)
Positive	34 (9.5%)	153 (42.7%)	12 (7.1%)	77 (45.3%)	9 (12.3%)	34 (46.6%)	42 (36.5%)	13 (11.3%)
Fever, (%)								
≥37.3°C	122 (34.1%)	67 (18.7%)	53 (31.2%)	35 (20.6%)	26 (35.6%)	10 (13.7%)	36 (31.3%)	14 (12.2%)
<37.3°C	41 (11.5%)	128 (35.8%)	21 (12.4%)	61 (35.9%)	6 (8.2%)	31 (42.5%)	22 (19.1%)	43 (37.4%)
Cough,(%)								
Yes	139 (38.8%)	163 (45.5%)	12 (7.1%)	17 (10%)	26 (35.6%)	35 (47.9%)	9 (7.8%)	6 (5.2%)
No	24 (6.7%)	32 (8.9%)	62 (36.5%)	79 (46.5%)	6 (8.2%)	6 (8.2%)	49 (42.6%)	51 (44.3%)
Expectoration,(%)								
Yes	109 (30.4%)	151 (42.2%)	25 (14.7%)	23 (13.5%)	20 (27.4%)	30 (41.1%)	10 (8.7%)	17 (14.8%)
No	54 (15.1%)	44 (12.3%)	49 (28.8%)	73 (42.9%)	12 (16.4%)	11 (15.1%)	48 (41.7%)	40 (34.8%)
Hemoptysis,(%)								
No	132 (36.9%)	140 (39.1%)	61 (35.9%)	69 (40.6%)	22 (30.1%)	32 (43.8%)	39 (33.9%)	49 (42.6%)
Yes	31 (8.7%)	55 (15.4%)	13 (7.6%)	27 (15.9%)	10 (13.7%)	9 (12.3%)	19 (16.5%)	8 (7%)

The data of normal distribution were described as mean ± standard deviation (SD) compared by t test. Data that are not normally distributed were expressed as median (range interquartile) and analyzed using the rank-sum test.

WBC, white blood cell count; NEUT%, neutrophil ratio; LYMPH%, neutrophil to lymphocyte ratio; CRP, C-reactive protein; HGB, Hemoglobin; PLT, platelets; ALB, albumin; GLU, glucose.

### Constructed radiomics model

**Figure 1:**
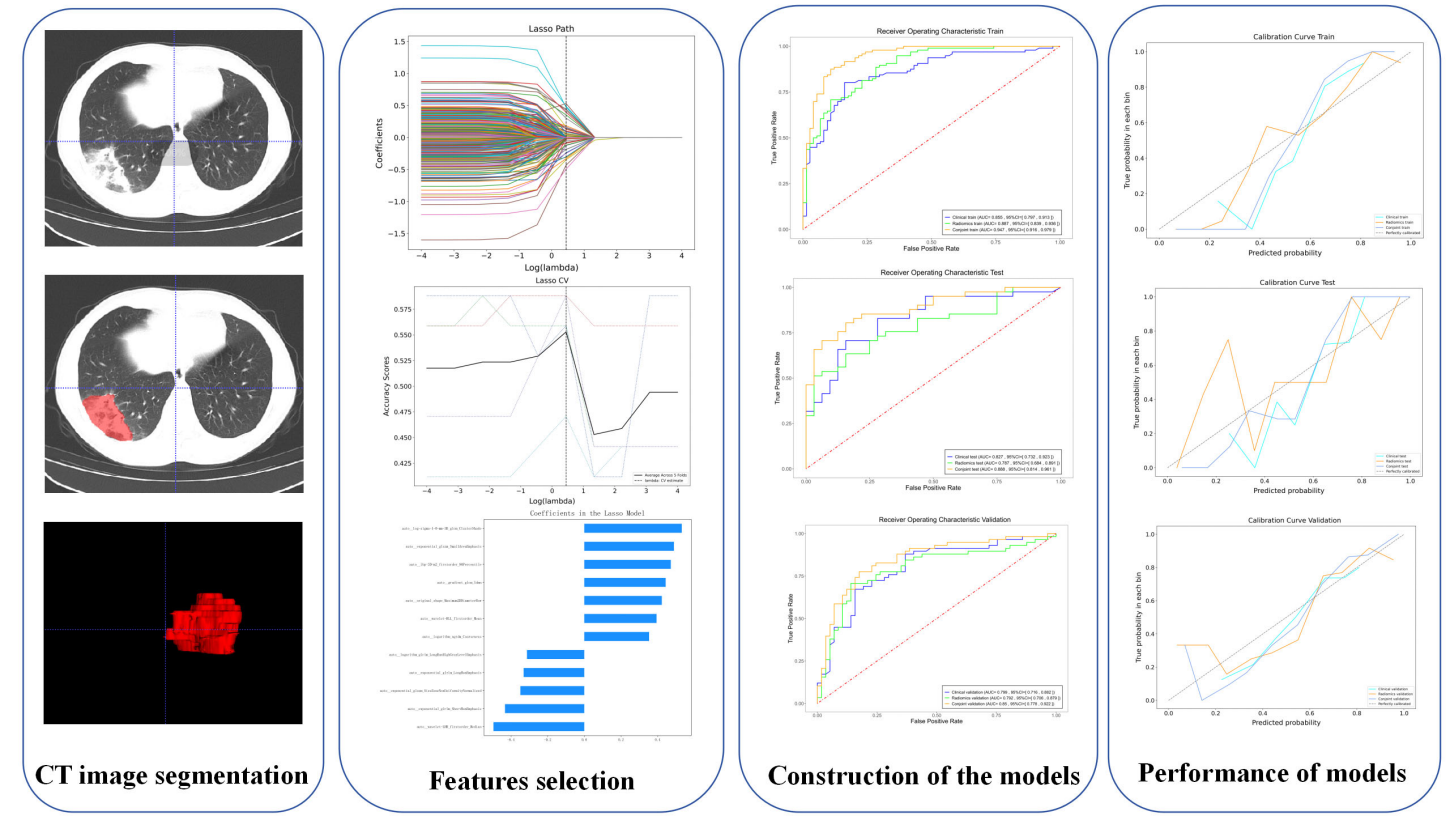
The radiomics flow chart of the study.

A total of 1557 radiomics features were extracted from each VOI, and a total of 12 different features were screened out by LASSO (**Table 2**). The details of the radiomics feature screening process are shown in **Figure 1**. The Radscore formula showed in Appendix S1.

**Table 2:** Radiomic Features Selection from the CT in the Training Cohort.

feature	Coefficient	p value
exponential_glrlm_LongRunEmphasis	-0.3308	0.0000
exponential_glrlm_ShortRunEmphasis	-0.4325	0.0000
exponential_glszm_SizeZoneNonUniformityNormalized	-0.3487	0.0343
exponential_glszm_SmallAreaEmphasis	0.4888	0.0000
gradient_glcm_Idmn	0.4440	0.0521
lbp-3D-m2_firstorder_90Percentile	0.4718	0.0010
log-sigma-1-0-mm-3D_glcm_ClusterShade	0.5316	0.0230
logarithm_glrlm_LongRunHighGrayLevelEmphasis	-0.3129	0.0000
logarithm_ngtdm_Coarseness	0.3535	0.0719
original_shape_Maximum2DDiameterRow	0.4228	0.1130
wavelet-HLL_firstorder_Mean	0.3940	0.0035
wavelet-LHH_firstorder_Median	-0.4954	0.0141

**Figure 2:**
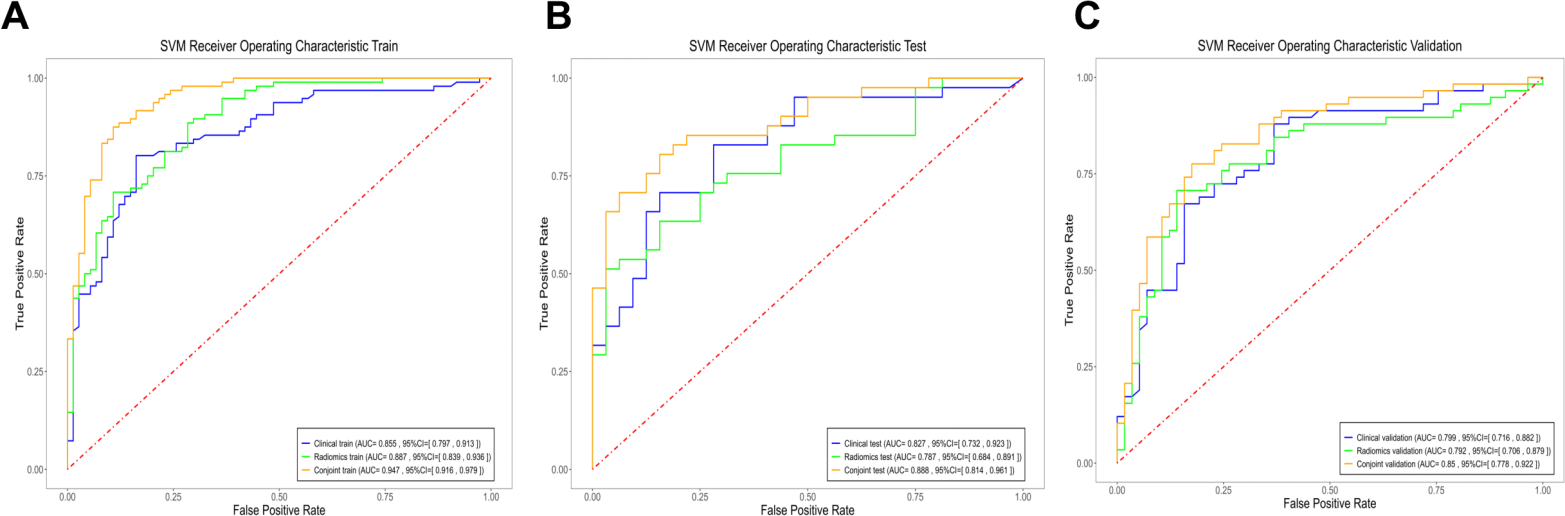
The ROC curves of the three models. **(A)** Three models of ROC curves in the training cohort. **(B)** Three models of ROC curves in the testing cohort. **(C)** Three models of ROC curves in the validation cohort.

In the identification of PTB and CAP in radiomics model, the AUC of the training cohort was 0.887 (95%CI: 0.839- 0.936), the AUC of the testing cohort was 0.787 (95%CI:0.684-0.891) and the AUC of the validation cohort was 0.792 (95%CI:0.706-0.879) (**Figure 2**).

### Establishment of a clinical radiomics conjoint nomogram

**Figure 3:**
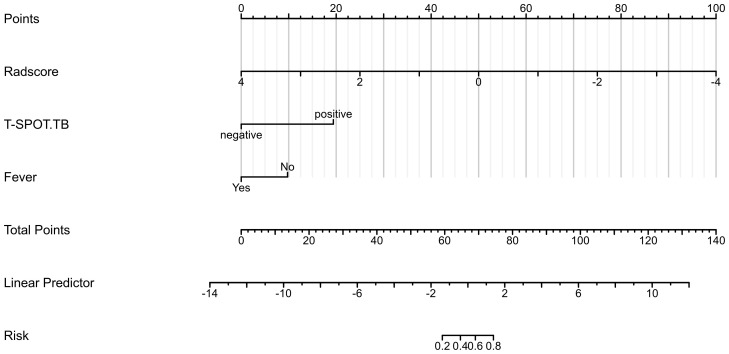
The clinical radiomics conjoint nomogram.

Based on the data of the training cohort, a clinical radiomics conjoint nomogram was established combined with the radiomics score, T-SPOT result, and fever, as shown in **Figure 3**. The C index of the nomogram is 0.955 (95%CI, 0.926 - 0.984).

### Assessment of the performance of the established model

**Figure 4:**
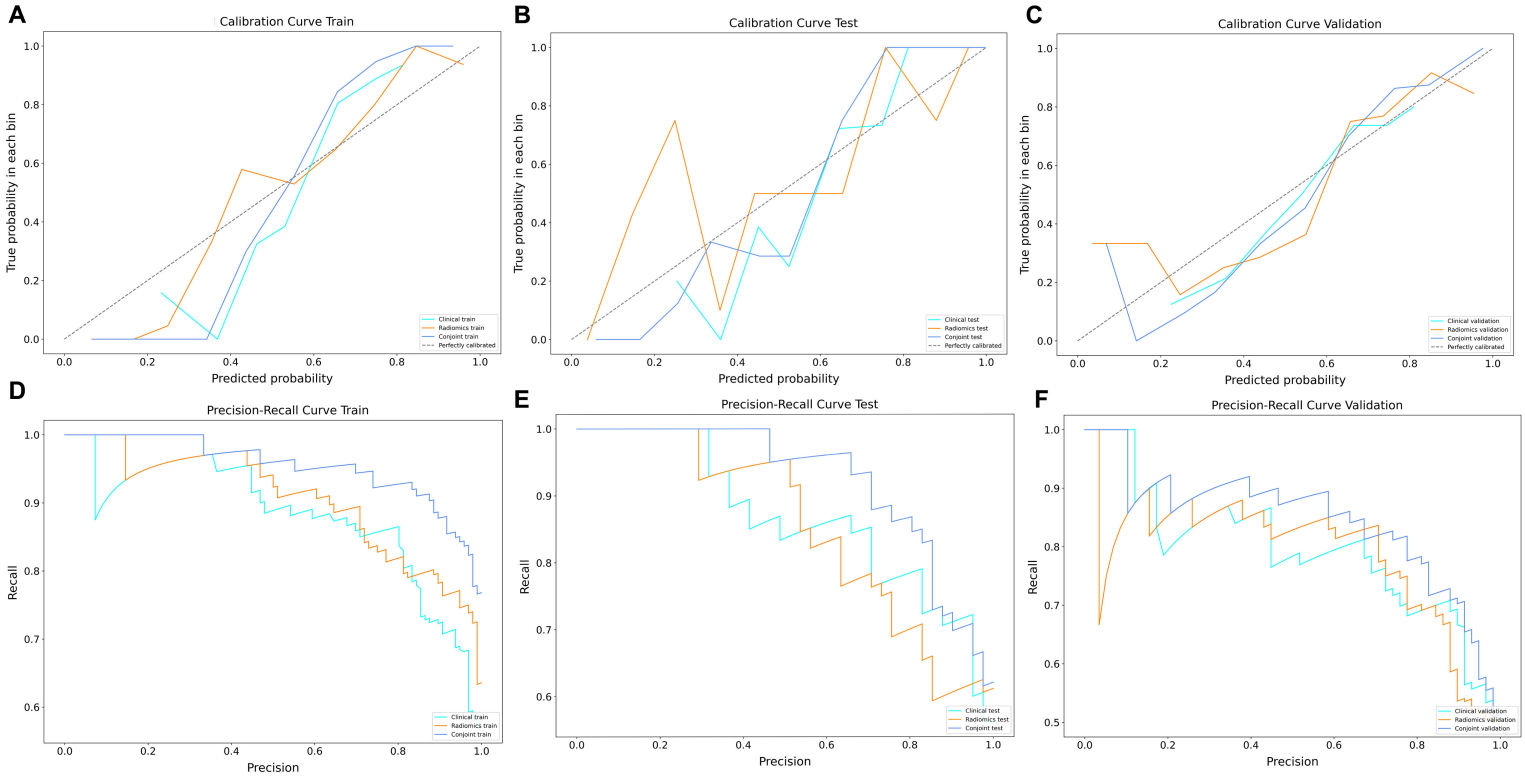
The decision curve and precision-recall curve analysis for three models. **(A)** Three models of decision curve in the training cohort. **(B)** Three models of decision curve in the testing cohort. **(C)** Three models of decision curve in the validation cohort. **(D)** Three models of precision-recall curve in the training cohort. **(E)** Three models of precision-recall curve in the testing cohort. **(F)** Three models of precision-recall curve in the validation cohort.

The results showed that the predictive nomogram had an outstanding ability to discriminate between the two diseases, with an AUC of 0.947 (95%CI, 0.916-0.979) on the training cohort, 0.888 (95%CI, 0.814-0.961) on the testing cohort and 0.850 (95%CI, 0.778-0.922) on the validation cohort. The discriminative power of the clinical model, radiomics model, and clinical radiomics model in the training and test cohorts is shown in **Figure 3**. The results showed that the clinical radiomics conjoint model was superior to the clinical model and the radiomics model. The calibration curve and precision-recall curve of the three models in the training cohort, test cohort and validation cohort are shown in **Figure 4**. **Table 3** provides a summary of the sensitivity, specificity, accuracy, positive predictive value, negative predictive value, false negative rate, false discovery rate, and false positives rate for all three models across the three cohort.

**Table 3:** Performance of the Clinical Model, Radiomics Model, and Clinical Model in the Training Cohort, Test Cohort and validation Cohort.

Different Models		AUC	SEN	SPE	ACC	F1	PPV	NPV	FNR	FDR	FPR
Radiomics Model	Train Cohort	0.887	0.885	0.716	0.812	0.842	0.802	0.828	0.115	0.198	0.284
	Test Cohort	0.787	0.756	0.563	0.671	0.721	0.689	0.643	0.244	0.311	0.438
	Validation Cohort	0.793	0.776	0.649	0.713	0.732	0.692	0.740	0.224	0.308	0.351
Clinical Model	Train Cohort	0.855	0.802	0.838	0.818	0.832	0.865	0.765	0.198	0.135	0.162
	Test Cohort	0.827	0.805	0.719	0.767	0.795	0.786	0.742	0.195	0.214	0.281
	Validation Cohort	0.799	0.707	0.772	0.739	0.732	0.759	0.721	0.293	0.241	0.228
Conjoint Model	Train Cohort	0.948	0.875	0.892	0.882	0.894	0.913	0.846	0.125	0.087	0.108
	Test Cohort	0.888	0.805	0.844	0.822	0.835	0.868	0.771	0.195	0.132	0.156
	Validation Cohort	0.850	0.759	0.825	0.791	0.786	0.815	0.771	0.241	0.185	0.175

AUC, the area under the curve; SEN, sensitivity; SPE, specificity; ACC, accuracy; PPV, Positive predictive value; NPV, Negative predictive value; FNR, False negative rate; FDR, False discovery rate; FPR, False positives rate.

## Discussion

At present, the clinical manifestations and CT imaging manifestations of PTB and CAP are similar. Clinically, it is difficult to distinguish between these two diseases. Previous studies have shown that solitary pulmonary tuberculosis and lung cancer can be effectively distinguished by combining CT imaging features and clinical variables (Yang et al., 2018; Feng et al., 2020; Hu et al., 2021). However, there are few studies on using radiomics to identify PTB from CAP. Our study used the methods to develop a model to more accurately identify PTB from CAP. Our results show that radiomic features can accurately differentiate between these two diseases both in a training and test cohort. Clinical radiomics nomogram can further improve its prediction efficiency, and DCA confirmed its clinical application value.

China is a region with high incidence of PTB, and the clinical manifestations of PTB are very similar to those of CAP. Moreover, CT findings of PTB are varied (Skoura et al., 2015). Typical CT findings include central lobular nodules, cavities, calcification, and caseous necrosis (Wang et al., 2021). However, an increasing number of patients with PTB have atypical manifestations, often presenting only as lung consolidation or ground glass shadow, which is easily confused with CAP (Yeh et al., 2014; Zeng et al., 2021). But the treatment is completely different. Clinically, the failure to identify PTB patients and CAP patients at an early stage is often one of the causes of tuberculosis transmission.

To distinguish PTB from CAP, we observed in univariate analysis of clinical characteristics that male, T-SPOT positivity, absence of fever, hemoptysis, and increased lymphocyte percentage were more common in patients with tuberculosis, consistent with previous findings (Jolobe, 2012; Yoon et al., 2013; Cavallazzi et al., 2014b; Cavallazzi et al., 2014a). Multivariate logistic analysis showed that among clinical factors, T-SPOT results and fever could be used as independent predictors to distinguish these two diseases. The T-SPOT assay is an interferon (IFN)-γ release assay. It is based on detecting secreted IFN-γ in M. tuberculosis-specific T-cells stimulated by Mycobacterium-specific antigens (Ma et al., 2019). Previous studies have shown that T-SPOT results have some power in the diagnosis of active pulmonary tuberculosis, but results are also affected by the state of the subjects (Zhu et al., 2014; Uda et al., 2020). Fever, especially hyperpyrexia, is more common in patients with CAP. Patients with PTB usually present with low-grade fever or even most do not present with fever (Chon et al., 2013), which is consistent with our findings. But on the whole, the clinical manifestations of the two diseases are similar, but the drugs used for treatment are different.

Our results revealed 12 radiomic features that differed significantly between PTB and CAP. These features typically capture texture variations to quantify the spatial relationships of voxels in an image (Pantic et al., 2014; Rios Velazquez et al., 2017). It is difficult for radiologists’ eyes alone to find these changes, so radiomic features can pick up subtle changes that are not discernible to the naked eye.

Therefore, we combined T-SPOT results and fever with the Radscore of 12 radiomics features selected by us to establish a clinical radiomics conjoint model in the training cohort. At the same time, the performance of the model is verified in the test cohort and the validation cohort respectively. The results showed that the clinical radiomics conjoint model is more advantages than the single radiomics model and clinical model. This indicates that the application of the clinical radiomics model is an efficient method that can help clinicians accurately distinguish PTB from CAP at an early stage.

There are some limitations in our study. First, this study is a retrospective study, and the error caused by confounding factors cannot be controlled. Secondly, although the study included three different medical centers, the overall sample size was small. Finally, the nomogram works best with linear models. If the model was complicated and non-linear, a standard nomogram would result in error. Therefore, we look forward to conducting a large prospective study to validate our preliminary results.

## Conclusions

In conclusion, this study developed a clinical radiomics conjoint model that uses radiomics features to identify PTB from CAP. Compared with the single radiomics model and clinical model, this model is more accurate, sensitive and specific. This model is of great value for the early detection, diagnosis and treatment of PTB.

## Data Availability

The original contributions presented in the study are included in the article/**Supplementary Material**. Further inquiries can be directed to the corresponding authors.
