# Supplementary material for: A CT-based radiomics predictive nomogram to identify pulmonary tuberculosis from community-acquired pneumonia: a multicenter cohort study
Source: Front Cell Infect Microbiol. 2024 Sep 19;14:1388991. doi: 10.3389/fcimb.2024.1388991 (PMC11446906; doi:10.3389/fcimb.2024.1388991)
Supplement: Supplementary file 1 [file Table1.docx]

Figure S1.The flow diagram of the study


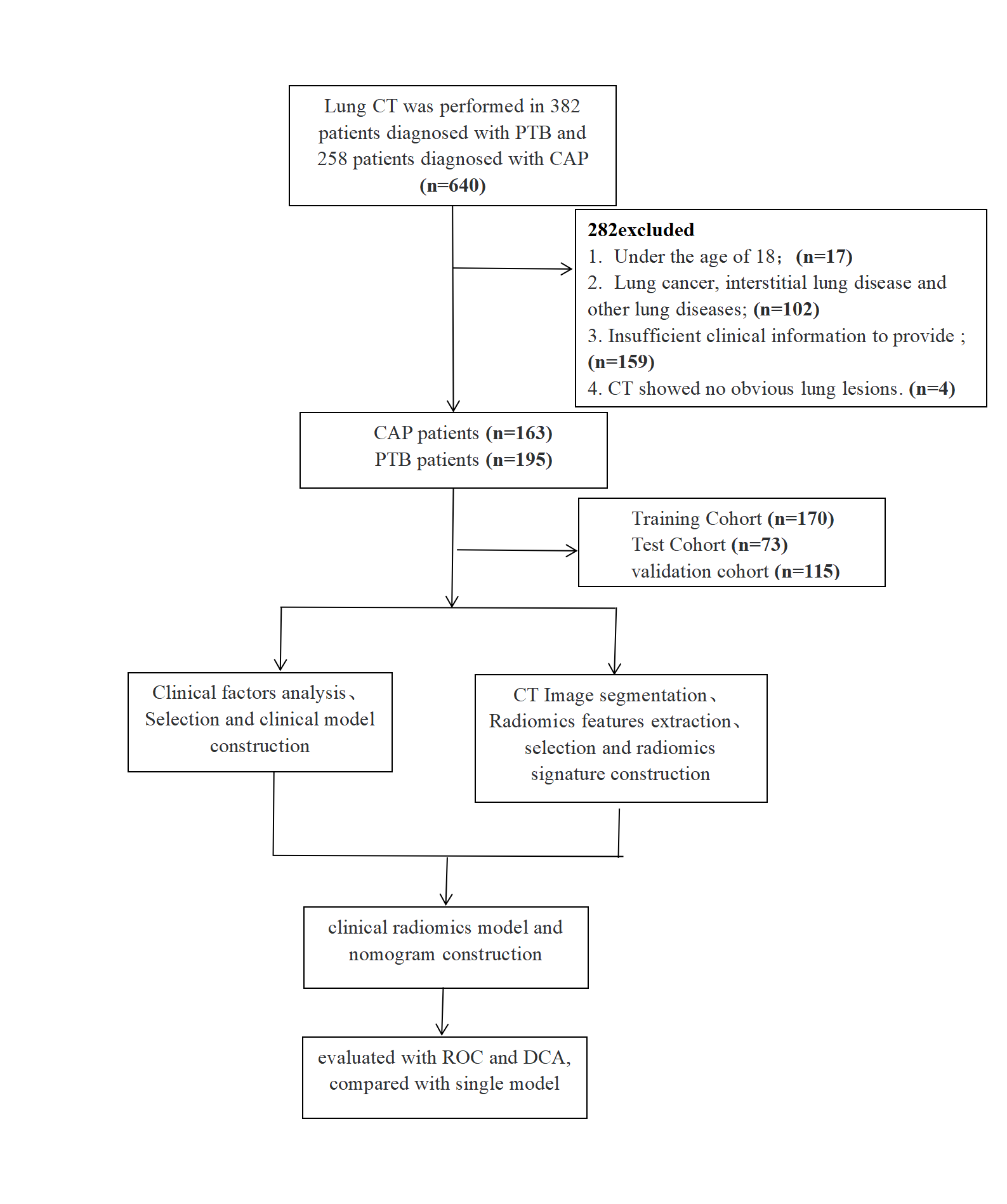


Appendix S1

Rascore = -0.3308325314195363 * exponential_glrlm_LongRunEmphasis - 0.4324805872196887 * exponential_glrlm_ShortRunEmphasis - 0.3486693011604215 * exponential_glszm_SizeZoneNonUniformityNormalized + 0.4888187334717249 * exponential_glszm_SmallAreaEmphasis + 0.4439903043329268 * gradient_glcm_Idmn + 0.4718158545267812 *lbp-3D-m2_firstorder_90Percentile + 0.531618540339001 *log-sigma-1-0-mm-3D_glcm_ClusterShade - 0.3129111533269489 * logarithm_glrlm_LongRunHighGrayLevelEmphasis + 0.3534518740902174 * logarithm_ngtdm_Coarseness + 0.4228230475182659 * original_shape_Maximum2DDiameterRow + 0.3940304361262208 * wavelet-HLL_firstorder_Mean - 0.4953514359829447 * wavelet-LHH_firstorder_Median

Table S1. The CT protocols of the three hospitals.

| Hospital | Machine Brand | Tube voltage (kV) | Tube current (mA) | Rotation time (s) | layer thickness (mm) | layer spacing (mm) |
| --- | --- | --- | --- | --- | --- | --- |
| Hospital I | General Electric, GE® (Optima CT540) ® | 120 | 160 | 0.5 | 5 | 5 |
| Hospital II | Philips (Ingenuity Core) ® | 100 | 150 | 0.5 | 5 | 5 |
| Hospital III | General Electric, GE® (Optima CT660) | 120 | 150 | 0.5 | 5 | 5 |

**Table S2.** Univariate and multivariate analysis of clinical variables.

|  | Univariate analysis | | Multivariate analysis | |
| --- | --- | --- | --- | --- |
| Characteristics | OR(95% CI) | p value | OR(95% CI) | p value |
| Sex |  |  |  |  |
| Female | Reference |  | Reference |  |
| Male | 1.640 (1.078 - 2.496) | 0.021 | 1.395 (0.759 - 2.561) | 0.284 |
| Age | 1.023 (1.011 - 1.034) | < 0.001 | 1.012 (0.996 - 1.028) | 0.154 |
| WBC | 1.084 (1.009 - 1.164) | 0.027 | 0.940 (0.825 - 1.072) | 0.356 |
| NEUT% | 1.042 (1.024 - 1.061) | < 0.001 | 0.980 (0.911 - 1.054) | 0.582 |
| LYMPH% | 0.947 (0.928 - 0.966) | < 0.001 | 0.935 (0.859 - 1.018) | 0.122 |
| HGB | 0.983 (0.973 - 0.993) | < 0.001 | 0.997 (0.985 - 1.009) | 0.637 |
| PLT | 1.005 (1.002 - 1.007) | < 0.001 | 1.006 (1.002 - 1.010) | 0.052 |
| ALB | 0.915 (0.882 - 0.950) | < 0.001 | 1.029 (0.969 - 1.093) | 0.349 |
| GLU | 0.971 (0.879 - 1.072) | 0.557 |  |  |
| CRP | 0.999 (0.995 - 1.002) | 0.477 |  |  |
| T-SPOT |  |  |  |  |
| negative | Reference |  | Reference |  |
| Positive | 13.821 (8.306 - 22.999) | < 0.001 | 12.377 (6.740 - 22.730) | < 0.001 |
| Fever |  |  |  |  |
| ≥37.3℃ | Reference |  | Reference |  |
| ＜37.3℃ | 5.685 (3.585 - 9.014) | < 0.001 | 5.010 (2.756 - 9.105) | < 0.001 |
| Cough |  |  |  |  |
| Yes | Reference |  |  |  |
| No | 1.137 (0.639 - 2.022) | 0.662 |  |  |
| Expectoration |  |  |  |  |
| Yes | Reference |  | Reference |  |
| No | 0.588 (0.368 - 0.939) | 0.026 | 0.883 (0.446 - 1.747) | 0.720 |
| Hemoptysis |  |  |  |  |
| No | Reference |  | Reference |  |
| Yes | 1.673 (1.014 - 2.759) | 0.044 | 1.129 (0.560 - 2.275) | 0.734 |

*WBC, white blood cell count; NEUT%, neutrophil ratio; LYMPH%, neutrophil to lymphocyte ratio; CRP, C-reactive protein; HGB, Hemoglobin; PLT, platelets; ALB, albumin; GLU, glucose.
